# Data-Driven Fundraising: Strategic Plan for Medical Education

**DOI:** 10.2196/53624

**Published:** 2024-07-22

**Authors:** Alireza Jalali, Jacline Nyman, Ouida Loeffelholz, Chantelle Courtney

**Affiliations:** 1 Faculty of Medicine, University of Ottawa, Ottawa, ON, Canada; 2 Telfer School of Management, University of Ottawa, Ottawa, ON, Canada; 3 Faculty of Health Sciences, University of Ottawa, Ottawa, ON, Canada

**Keywords:** fundraising, philanthropy, crowdfunding, funding, charity, higher education, university, medical education, educators, advancement, data analytics, ethics, ethical, education, medical school, school, support, financial, community

## Abstract

Higher education institutions, including medical schools, increasingly rely on fundraising to bridge funding gaps and support their missions. This paper presents a viewpoint on data-driven strategies in fundraising, outlining a 4-step approach for effective planning while considering ethical implications. It outlines a 4-step approach to creating an effective, end-to-end, data-driven fundraising plan, emphasizing the crucial stages of data collection, data analysis, goal establishment, and targeted strategy formulation. By leveraging internal and external data, schools can create tailored outreach initiatives that resonate with potential donors. However, the fundraising process must be grounded in ethical considerations. Ethical challenges, particularly in fundraising with grateful medical patients, necessitate transparent and honest practices prioritizing donors’ and beneficiaries’ rights and safeguarding public trust. This paper presents a viewpoint on the critical role of data-driven strategies in fundraising for medical education. It emphasizes integrating comprehensive data analysis with ethical considerations to enhance fundraising efforts in medical schools. By integrating data analytics with fundraising best practices and ensuring ethical practice, medical institutions can ensure financial support and foster enduring, trust-based relationships with their donor communities.

## Introduction

Higher education institutions play a crucial role in shaping society’s future by developing new ideas, advancing knowledge, and preparing future leaders. However, to fulfill this role, institutions need financial resources. Digital medicine, a rapidly evolving field of technology and health care, is revolutionizing how fundraising is conducted toward funding medical research and public health initiatives. Data, in conjunction with artificial intelligence, are transforming health care and paving the way for personalized medicine. By leveraging cutting-edge digital tools in data analysis, organizations can now gather, process, and interpret vast amounts of health-related data more efficiently [1]. This data-driven approach enables a deeper understanding of disease patterns, patient needs, and effective treatment strategies, facilitating more targeted and impactful fundraising efforts (by segmenting fundraising strategies in keeping with these new patient data streams). Furthermore, innovative fundraising platforms are emerging, harnessing the power of artificial intelligence and algorithmic data analysis that draws from and informs social media, mobile technology, and web-based crowdfunding [2]. These platforms expand the reach of fundraising campaigns and allow for real-time engagement with donors, aimed at enhancing transparency and trust. By integrating these digital advancements, fundraising with digital medicine is becoming more efficient and more personalized, aligning donor interests with specific health care projects and research endeavors.

Fundraising is vital to higher education because it helps organizations acquire some of the financial resources needed to achieve their mission, vision, and strategic goals. Fundraising can help institutions fund new academic programs, foster research and student learning, advance knowledge, and build links with the community [3,4]. Higher education and medical schools, in particular, are expensive [5], and many students require financial assistance to attend college or university. Fundraising can help institutions provide financial support for scholarships, grants, and other forms of financial aid to students who need it most. The paper is aimed at a novice audience, providing a basic framework for data-driven fundraising. It is set in a Canadian context, where resources such as wealth indicators are less prevalent than those in the United States.

Proceeds generated from fundraising endeavors can enhance and modernize campus infrastructure, encompassing vital domains such as classrooms, laboratories, libraries, dormitories, and athletic amenities. Increased financial resources from fundraising activities can be strategically allocated to recruiting and retaining distinguished faculty and researchers, establishing scholarships and awards for students, and supporting community outreach activities.

To provide a broader context on fund development terminology, it is crucial to differentiate and link fund development, fundraising, and advancement:

- Fund development: This term encompasses the overarching process of creating and nurturing relationships that foster an organization’s growth. It includes strategic planning, donor engagement, and stewardship, going beyond mere transactional activities to build a sustainable funding base.
- Fundraising: This is a subset of fund development focused on securing funds. It may involve direct or web-based solicitation, single-purpose or multiyear comprehensive campaigns, events, grant writing, and sponsorships. Fundraising is the operational action that stems from the broader fund development strategy.
- Advancement: In academic and nonprofit sectors, advancement refers to efforts that advance the institution’s mission through fundraising, stakeholder or alumni relations, public affairs, and marketing.

Advancement offices play a pivotal role in the ecosystem of fundraising and donor engagement within institutions. They are responsible for leading an ongoing dialogue between the faculty and the prospective or current donors. Key to advancement work is seeking to understand the impact that the donors want to make through their philanthropic activities and connecting those goals with approved fundraising priorities when possible. Furthermore, advancement offices are instrumental in identifying and capitalizing on various funding opportunities, ensuring that the institution’s projects and initiatives are adequately supported. They also provide crucial support for single-purpose campaigns, offering strategic guidance and resources to shape success. By doing so, these offices foster immediate financial support and build long-term relationships between the institution and its benefactors.

Universities increasingly establish “advancement” units integrating fund development, fundraising, alumni, and community outreach activities. The term “fund development” is preferred, as it emphasizes cultivating and maintaining long-term relationships and support. This approach is integral to fostering enduring commitments rather than transactional contributions. Within universities, fundraising can occur at various levels, including within smaller units, departments, or faculties, as well as at the university-wide level. However, consulting and collaborating with advancement or fund development professionals are crucial before initiating fundraising efforts. This ensures that fundraising activities are in harmony with the broader institutional plans and goals, as well as coordinated among the different units and subunits to ensure maximum impact.

Professionals in advancement offices play a pivotal role in managing potential donor relationships, understanding their interests, and aiding units in crafting strategic fundraising plans and proposals. They are skilled in identifying donor interests in the institution and its programs, often starting with smaller initiatives to support donor engagement. These experts often keep a comprehensive list of funding opportunities, including scholarships, bursaries, and specific project or equipment funding. They may support small-scale or single-purpose campaigns for various university needs, such as funding a simulation center or a theater. Advancement professionals also know about the institution’s approach to endowment funds versus expendable or “flow-through” funds. Endowments are typically maintained in perpetuity, providing a unit with a percentage of the total endowed principal plus investment, whereas expendable funds are designated for use over a predetermined period until depleted. Advancement professionals’ expertise is vital in aligning donor intentions with the vision, strategic plan, and priorities of the university. Furthermore, these fundraising professionals are known to play the role of networker, negotiator, and knowledge broker [6].

The aim of this paper is to explore a data-driven approach to fundraising in medical education, integrating comprehensive data analysis with ethical considerations.

## A Fundraising Road Map

A fundraising plan or road map is aligned with an institution’s strategic plan and is an essential component of a successful fundraising effort. It is a written document that sets Specific, Measurable, Achievable, Relevant, and Time-bound (SMART) goals [7] for the fundraising campaign and communicates these goals and objectives to internal and external stakeholders, including faculty, staff, donors, prospective donors, and the general public. The road map helps determine the resources (human, financial, material, etc) needed to successfully implement the campaign, including academic leadership, advancement staff, volunteers, campaign materials, and so on, as well as how best to allocate these resources to maximize the return on investment (ROI).

Establishing SMART objectives is crucial for the success of fundraising campaigns. These objectives provide a clear road map, ensuring that every aspect of the fundraising effort is intentional and efficient. A well-structured fundraising plan, guided by these SMART objectives, allows for a strategic approach, setting clear milestones and measurable goals. This approach facilitates focused efforts, efficient resource allocation, and the ability to track progress effectively. Such meticulous planning is essential for aligning the fundraising activities with the institution’s overarching goals and ensuring the optimal use of resources for maximum impact. Although setting SMART objectives is foundational, the consistent application of workflows, standards, and daily execution ultimately determines the campaign’s success.

The plan also aims to recognize the organization’s most generous philanthropic contributors. In particular, it helps ensure that prospective supporters with the necessary financial capacity are engaged, including alumni and friends, corporations, foundations, and government agencies, and that they share an interest in furthering the organization’s mission, strategic goals, and fundraising priorities. The road map often includes targeted cultivation, solicitation, and stewardship strategies by fundraising program, unit, geographic region, and source of funds (eg, cash, gifts of publicly traded securities) to ensure maximum ROI [6].

By establishing a framework that includes timelines for evaluating the campaign’s success, the fundraising plan helps keep the campaign on track by checking the progress made against goals set on a monthly, quarterly, and annual basis. The plan also includes key performance indicators to measure each fundraising program and solicitor’s success. Some key performance indicators may consist of the number and quality of meetings undertaken with potential donors, gift proposals submitted, gifts received, total funds raised, number of new donors, and gift size; ultimately, they impact the organization.

Fundraising activities should align strategically with an institution’s broader goals and plans. This alignment ensures that fundraising efforts secure the necessary funds and support and advance the institution’s mission and objectives. The fundraising plan is closely aligned with the institution’s strategic priorities, ensuring that fundraising supports key teaching, learning, and research goals, establishing a long-term focus instead of a short-term financial solution. Collaborating with advancement and fund development professionals is crucial in this process. These experts bring invaluable insights and strategies that help identify and engage with potential donors whose interests and values resonate with the institution’s goals. Such collaboration ensures that fundraising activities are not only successful in the short term but also contribute to the long-term growth and success of the institution.

## Using Data in Fundraising

Data play a critical role in developing a successful fundraising plan. Data provide valuable insights into the past, current, and future donor areas of philanthropic interest and a measurement of the effectiveness of past fundraising campaigns, strategies, programs, and methods. Once analyzed, data can guide the fundraising efforts toward opportunities for growth and help offer the indicators of potential risks or barriers to success. Publicly available data can help drive donor strategies in keeping with their consumption interests. Algorithms are used to make sense of these masses of data and to help predict the greatest potential fundraising strategies and potential donors.

Medical schools (or any faculty) can develop a data-driven fundraising plan based on information and insights aligned with achieving their mission and fundraising goals. Using data to inform strategy and decision-making, medical schools can develop more effective and targeted fundraising plans that deliver better results and build stronger relationships with donors, prospective donors, and the intended beneficiaries [8].

Establishing a data-centered culture in fundraising involves a strategic shift toward using data analytics to guide decision-making processes. This approach emphasizes collecting, analyzing, and leveraging data to understand donor behaviors, preferences, and trends. By adopting a data-centered culture, institutions can make informed decisions about whom to approach, when, and how, thereby increasing the effectiveness and efficiency of their fundraising efforts. Guidance on this aspect includes investing in the right tools and training for data analysis, cultivating a mindset among staff that values data-driven insights, and continuously refining strategies based on data feedback.

Making the data actionable is crucial; it should directly inform and shape your fundraising strategies. MacLaughlin [9] highlights the importance of using data strategically in fundraising. Key points include prioritizing data quality, using data for effective segmentation and personalized communication, and fostering a culture of experimentation. Data should inform strategies, help identify potential donors, and predict giving patterns. Investment in data skills and tools is crucial for effective analysis. Data-driven storytelling can demonstrate donation impact, while good data governance ensures responsible data management. A growth mindset encourages learning from successes and failures. Understanding the difference between business intelligence (analyzing historical data) and predictive analysis (using data to predict future outcomes) can also enhance fundraising strategies. While this paper focuses on business intelligence, which involves diagnostic analytics, predictive analysis involves forecasting future outcomes and represents a more advanced stage of data-driven fundraising.

## The 4 Pillars of Data-Driven Fundraising

There are four key steps to developing an end-to-end data-driven fundraising plan: (1) data collection; (2) data analysis; (3) establishing fundraising goals and objectives; and (4) formulating targeted fundraising strategies.

### Data Collection

The first step for medical schools in developing a data-driven fundraising plan is collecting quantitative and qualitative data. External and internal research, both from primary and secondary sources, are critical. External information about current and potential donors is often publicly available, including secondary data sets such as demographic and career information, professional networks, and affiliations; giving history; and philanthropic interests. Institutional primary donor data can be analyzed to determine linkages to the medical school; affinity; and giving patterns, including gift amounts, giving frequency, past gift designations, and retention rates. These primary data sets can often be used in algorithmic analysis (in combination with secondary data available in the marketplace) to deepen our understanding of the donor landscape.

Additional information about donor experience, potential future giving interests, and preferred methods of communication can be gleaned from various data collection tools, such as surveys, focus groups, personal meetings, and social media. Internal and external data sets, as well as primary and secondary data sets, can be used together and separately to help organizations better understand donors’ and prospective donors’ interests, as well as the financial capacity and emotional links to the organization, thereby enabling medical schools to develop targeted outreach strategies that better meet donors’ needs and preferences.

In addition to collecting data on donors, it is also important to have access to information about an institution’s past fundraising campaigns, comparative data about other professional schools and peer organizations, and industry trends. These data provide benchmarks and guideposts for planning, measurement, and evaluation.

### Data Analysis

Once data on donors, past campaigns, and industry trends have been collected, they can be analyzed to uncover patterns and trends that provide insights for future fundraising goals, objectives, strategies, and resource allocation. For example, by examining the giving patterns of alumni, friends, retirees, corporations, and foundations, analysts can pinpoint the organization’s foremost donors in terms of total contributions. Data analysis can reveal which donors have made the most substantial gifts, including those with deep ties to the organization, based on their motivations, giving history, donation frequency, level of involvement, and satisfaction. Based on these data points, predictive analysis can help guide donor stewardship and retention strategies, as well as determine which donors are most likely to increase their contributions, both in terms of their capability and willingness to give more. Analysis allows fundraising campaign planners to highlight individuals and organizations currently not donating but with significant potential to support crucial future initiatives.

Data from past fundraising campaigns can be analyzed to determine what programs (annual, major, principal, and planned gifts) and strategies were the most successful against their respective goals. Analysis helps us understand performance trends (high and low) and which areas have the most potential for future growth. Similarly, fundraising tactics, such as direct mail, call centers, electronic solicitations, personal approach, social media, events, and so on, can be evaluated to see which methods have the greatest ROI and growth potential. Understanding that donor-acquisition methods are generally costlier than those used to upgrade existing donors, steps in donor acquisition, retention, and upgrading can be further analyzed to understand and guide the fundraiser’s strategic planning and resource deployment. Data can also help determine whether some geographical regions, sources of revenue, fund designations, cohorts, and programs will provide the most predictable ROI for scarce faculty resources.

An analysis of data on current and prospective donors; past campaign successes and failures; peer organizations; and industry, socioeconomic, and technological trends are some of the factors to consider when determining where to allocate future resources for maximizing fundraising success. A thoughtful fundraising strategy can also further equity, diversity, and inclusion initiatives, inviting a broad range of Canadians and international citizens to invest in specific educational missions.

### Establishing Fundraising Goals and Objectives

To establish an overall fundraising campaign with SMART goals, medical schools should start by determining the cost of delivering programs and services as per their strategic plan and expected revenue from nonfundraising sources. Once the revenue gap is determined, fundraising campaign planners can then examine the following: (1) past fundraising campaign results; (2) financial capacity and affinity as well as inclination of the faculty’s donor pool, notably high–net worth individuals with the greatest capacity to give; (3) opportunities for growth based on institutional, industry, technological, and global trends; (4) social, economic, and political climate; (5) organizational reputation; and (6) potential risks. These can be used to discover a realistic overall fundraising goal depending on the time frame established.

However, of utmost importance is establishing a compelling case in support of a given program or project (gift designation). Donors give because they are deeply interested in supporting a given cause and because of the impact their gift will have on the beneficiaries. Research on high–net worth donors has concluded that donors give because they want to make transformational change, have a societal impact, and leave a personal legacy [10]. So, as we analyze the data to focus on our most promising potential donors and the most efficient fundraising strategies, a compelling case for support remains crucial to the success of any fundraising program.

In addition to the overarching fundraising goal, there are other subgoals that organizations can establish to align with the overarching goal. For example, the subgoals may be to increase the total number of donors to a medical school by a specific percentage (ie, the percentage of alumni who give back to their alma mater), as well as increase the average gift size per donor, the number of annual campaign donors upgraded to major gifts, the number of new monthly donors, and the number of new multiyear pledges. Subgoals are chosen from the data analysis as these data points align with increasing overarching fundraising success.

Subgoals can also be established by year or decade of graduation; region; fundraising program (eg, an annual campaign, major gifts, principal gifts, and planned giving); source (eg, alumni, friends, foundations, corporations, and other organizations); gift designation (eg, case for supporting students, research, infrastructure, and community engagement); and individual fundraiser or solicitor, team, and unit. Donor communication, engagement, and stewardship subgoals can also be set. Aligning subgoals and measurements with overarching goals associated with success metrics supports an integrated strategy.

### Developing Targeted Fundraising Strategies

Once SMART fundraising goals have been established, medical schools can use data to develop targeted strategies and related activities, budgets, and timelines to reach their organizational goals and objectives.

For example, if the goal is to increase revenue to a specific fund designation or case for support, and the data reveal specific donor groups to be most supportive of these, then targeted communications, outreach, and solicitation strategies can be developed to better reach these donor groups, thereby increasing the chances of fundraising success.

For example, suppose past giving trends indicate that alumni are most likely to support scholarship funds. In that case, medical schools may focus their fundraising efforts and activities on reaching out to their alumni via various means of communication, highlighting the impact that scholarships have on student access and achievement, and inviting alumni to make or upgrade their commitment to scholarship funds. In other words, the fundraising ROI becomes greater by reinforcing the compelling case for support with donor groups most likely to respond.

In another example, if the data show that a certain percentage of a medical school’s donors is in the 60+ years age range, the faculty can develop focused, planned giving strategies, communicating how legacy gifts (eg, deferred giving in the form of a bequest or life insurance) contribute to the long-term sustainability of the medical school’s vision. If the data show that a significant number of high–net worth donors live in a particular geographical area, regional engagement, cultivation, and solicitation strategies can be developed accordingly better to meet the needs and interests of this group. This list of examples shows how targeted approaches can be developed through data analysis and planning.

## Ethical Practice in Fundraising

Ethics in higher education fundraising are about conducting fundraising efforts in a manner that is consistent with ethical values and principles and that fosters trust and accountability. In a normative context, it can be said that “fundraising is ethical when it promotes and protects trust in fundraising and unethical when it harms trust.” [11] Medical schools must ensure that fundraising practices are transparent, honest, and responsible, and that all parties involved in the fundraising process are treated with respect.

Ethical practices in fundraising are of utmost importance, particularly in maintaining trust and integrity in the relationship between institutions and donors. A critical aspect of this is developing and adhering to clear gift acceptance policies. These policies help ensure that all donations are aligned with the institution’s mission and ethical standards. In addition, it is crucial to manage the extent of donor influence. While donor engagement is important, institutions must maintain autonomy and ensure that donations do not compromise their values or objectives. Upholding these ethical standards fosters a transparent and trustworthy environment and protects the institution’s reputation and long-term sustainability.

Many fundraisers in Canada are guided by the Association of Fundraising Professionals Code of Ethical Principles, which encourages them to practice their profession with integrity, honesty, and truthfulness, as well as safeguard public trust [12]. The Association of Fundraising Professionals Donor Bill of Rights highlights the principle of philanthropy rooted in voluntary action for communal benefit, emphasizing transparency, trust, and responsible stewardship in nonprofit engagements. It outlines donors’ rights, including being informed of the organization’s mission, usage of donations, board identity, access to financial statements, assurance of gift use as intended, respectful and confidential handling of donation information, professional interactions, clarity on the status of solicitors (volunteers or employees), option to opt out from mailing lists, and the freedom to inquire and receive honest responses when donating [13].

The authors make the case that “fundraisers are unlike commercial marketers in that they arguably have two key constituencies—their donors and their beneficiaries through a transfer rather than an exchange” [11]; therefore, there must be balance in protecting both donors and beneficiaries and not applying ethics to one at the expense of another.

Fundraisers should be transparent about the intended purpose of the funds being raised, how the funds will be used, and any potential benefits or risks associated with donating. Furthermore, they should treat all potential donors and beneficiaries fairly and equitably, without discrimination or favoritism. Donors’ data, including personal information and donation history, should be confidential and not shared with third parties without consent [8]. Moreover, fundraisers should avoid situations that could create conflicts of interest for themselves, their organizations, the donors, and the beneficiaries.

One area of importance is grateful patient fundraising (GPFR), a unique approach to charitable giving where patients, often touched deeply by the care they have received, choose to support their health care institutions financially. Although GPFR is widespread, it raises ethical issues for patients, physicians, development professionals, and institutions. In 2004, the American Medical Association Council on Ethical and Judicial Affairs acknowledged that philanthropic donations are essential to maintaining state-of-the-art medical facilities and conducting research. However, they discouraged physicians from directly soliciting from their patients, especially during a clinical encounter [14]. More recently, Collins et al [13] made a list of recommendations and stated, among others, that GPFR discussions must be avoided when patients are clinically vulnerable. Philanthropy does not justify a level of medical care not available to other patients, and institutions should recognize and take measures to mitigate the ethical risks inherent in wealth screening [15].

Educational institutions are also responsible for using donated funds wisely and effectively and ensuring that the intended purposes of the donations are fulfilled. Institutions are urged to have a fundraising committee of educators, physicians, and fundraisers to reduce safety concerns and prevent fraudulent behavior. It is crucial to establish clear gift acceptance policies and set limitations on donor influence to maintain transparency and ethical fundraising practices. Fundraisers are required to abide by the established rules of their fundraising organization [16].

## Conclusions

Developing a fundraising plan or road map is crucial for medical education institutions to achieve their institutional vision and goals; allocate resources effectively; and raise the funds they need to make up for budget shortfalls, remain competitive, and transform their institutions. This is a partnership among the advancement office, practitioner fundraisers, and academic leadership.

A fundraising plan with clear goals, objectives, strategies, action plans, and timelines helps build stronger relationships with existing and prospective donors and volunteers by providing them with a clear understanding of the institution’s needs and goals, the institution’s plan to achieve these goals, and how their gifts can make a difference.

Data collection and analysis are essential for establishing SMART fundraising goals and developing strategies to yield the greatest results. By having a well-designed and data-driven fund development plan, medical schools can ensure that they have the resources to support their students, research, and mission in the short and long term.

## Abbreviations

GPFR: grateful patient fundraising
ROI: return on investment
SMART: Specific, Measurable, Achievable, Relevant, and Time-bound
